# Implications of land use change on the national terrestrial carbon budget of Georgia

**DOI:** 10.1186/1750-0680-5-4

**Published:** 2010-09-13

**Authors:** Pontus Olofsson, Paata Torchinava, Curtis E Woodcock, Alessandro Baccini, Richard A Houghton, Mutlu Ozdogan, Feng Zhao, Xiaoyuan Yang

**Affiliations:** 1 Department of Geography and Environment, Boston University, 675 Commonwealth Avenue, Boston, MA 02215, USA; 2 Vasil Gulisashvili Forest Institute, 9 Mindeli Street, Tbilisi 0186, Georgia; 3 Woods Hole Research Center, 149 Woods Hole Road, Falmouth, MA 02540, USA; 4 Department of Forest and Wildlife Ecology, University of Wisconsin-Madison, 1630 Linden Drive, Madison, WI 53706, USA

## Abstract

**Background:**

Globally, the loss of forests now contributes almost 20% of carbon dioxide emissions to the atmosphere. There is an immediate need to reduce the current rates of forest loss, and the associated release of carbon dioxide, but for many areas of the world these rates are largely unknown. The Soviet Union contained a substantial part of the world's forests and the fate of those forests and their effect on carbon dynamics remain unknown for many areas of the former Eastern Bloc. For Georgia, the political and economic transitions following independence in 1991 have been dramatic. In this paper we quantify rates of land use changes and their effect on the terrestrial carbon budget for Georgia. A carbon book-keeping model traces changes in carbon stocks using historical and current rates of land use change. Landsat satellite images acquired circa 1990 and 2000 were analyzed to detect changes in forest cover since 1990.

**Results:**

The remote sensing analysis showed that a modest forest loss occurred, with approximately 0.8% of the forest cover having disappeared after 1990. Nevertheless, growth of Georgian forests still contribute a current national sink of about 0.3 Tg of carbon per year, which corresponds to 31% of the country anthropogenic carbon emissions.

**Conclusions:**

We assume that the observed forest loss is mainly a result of illegal logging, but we have not found any evidence of large-scale clear-cutting. Instead local harvesting of timber for household use is likely to be the underlying driver of the observed logging. The Georgian forests are a currently a carbon sink and will remain as such until about 2040 if the current rate of deforestation persists. Forest protection efforts, combined with economic growth, are essential for reducing the rate of deforestation and protecting the carbon sink provided by Georgian forests.

## Background

The increase in atmospheric carbon dioxide is primarily a result of fossil fuel burning and conversion of land use, with one third of the increase since 1750 being attributed to land use change [1]. In its Fourth Assessment Report, The Intergovernmental Panel of Climate Change (IPCC) estimates that forest degradation and deforestation is now contributing almost 20% of the greenhouse gas emissions to the atmosphere [2]. Accordingly, there is an immediate need to reduce the current rates of forest loss and degradation, and the associated release of carbon dioxide. However, we continue to lose forest at an alarming rate - the net loss in global forest area between 2000 and 2005 was about 7 million ha per year, which is equivalent to a net loss of 20,000 ha of forest each day [3]. For humid tropical forests alone, 4.5 million hectares per year were deforested during the same time period according to remote sensing estimates [4]. Besides the associated emission of carbon to the atmosphere, loss and degradation of forest have severe impacts on biological diversity and local living conditions. The goal of the UN-REDD (United Nations Collaborative Programme on Reducing Emissions from Deforestation and Forest Degradation in Developing Countries), established in 2005, is to reduce green house gas emissions from deforestation while maintaining and improving sustainable management of forests [5]. Although deforestation is mainly a result of land being converted for agricultural purposes [6], the causes and nature of deforestation are often complex and may vary significantly from country to country. International efforts toward increasing sustainable forest management in developing countries have proven difficult, and the addition of reducing green house gas emissions adds another layer of complexity [5].

UN-REDD activities are currently confined to tropical countries but the issues of deforestation and degradation are also present in many of the transitional economies in the former Eastern Bloc. Temperate forests in these areas have received little attention and the effects of the political and economical transitions on forest cover and land use remain unclear to a large extent. Studies at the scale of countries are, for example, largely non-existent. Thus, there is a need for international efforts such as UN-REDD to expand the study of deforestation/degradation and land use change to include the temperate regions of transitional economies, as carbon emissions from forest loss in these areas will also influence climate.

Tracking of deforestation is mainly carried out using national official forest resource statistics [3,6]. Although this gives important information on the status on global forest resources; inconsistencies in methods and definitions, differences in drivers of forest transitions, and varying degrees of development between and within nations, raise questions on the reliability of forest statistics while highlighting the importance of remote sensing of forest resources [4,7-11].

Illegal logging adds further complexity to the issue. More than half of the harvested timber in certain South American, African and Southeast Asian countries is estimated to be illegal [9]. The World Bank estimates that illegal logging on public lands deprives developing countries of US$10 to US$15 billion annually. Further, illegal logging has far-reaching environmental, social and economic consequences [12]. Accurate estimates of illegal harvests are unlikely in the official resource statistics, and remote sensing is often the only feasible way to acquire reliable estimates of the total amount of cleared forest. This holds true for many parts of the former Eastern Bloc [7,13-15]. With the collapse of the Soviet Union in the beginning of the 1990 s, a radical transformation of the forest industry took place as centralized power was transferred to local institutions, ranging from local governments to small private enterprises [15]. The effect of this transition on land use change and related carbon dynamics remains unclear to a large extent.

**Figure 1:**
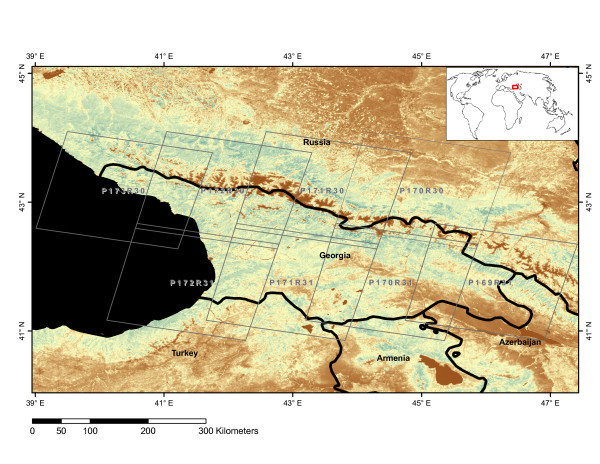
**Map of Georgia**. Terra/MODIS 250 m EVI with the international boundaries of Georgia and its neighboring countries; and the extent of the Landsat scenes used in the analysis. The scene extents are labelled with the WRS path and row number.

For Georgia (Figure 1), the changes following independence from the Soviet Union in 1991 were dramatic. Population and GDP dropped, and urban unemployment rose [16,17]. Although the country has experienced development since 1993, a majority of the Georgian households still relies on wood for fuel [18]. Situated on the Caucasus, Georgia is home to a diverse fauna and flora, and almost half the country is covered by forests. But as with many other former Soviet republics, the environmental implications of the social and political transitions after 1990 remain unclear. The nature and rate of land use change, deforestation and illegal logging, and the associated carbon dynamics remain uncertain. For these reasons, we attempt to quantify the rates of land use change and their effect on the terrestrial carbon budget for Georgia. Historical land use rates are obtained from the Georgian forest department whereas changes from 1990 to 2000 are estimated using data from the Landsat satellites. A carbon book-keeping model (or "carbon accounting model") is run using the obtained rates of land use change to estimate the associated carbon sinks and sources over time.

## Results

### Remote Sensing

**Figure 2:**
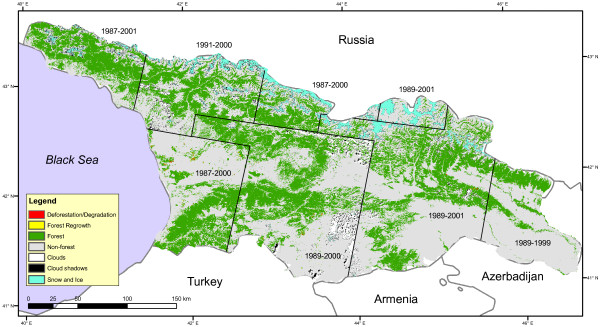
**Georgia changemap**. Changemap for Georgia, from circa 1990 to 2000. Red is forest loss during this time period, while green is stable forest (i.e. forest present in both 1990 and 2000). Gray is stable non-forest (i.e. non-forest present in both 1990 and 2000). The map also includes the forest regrowth (yellow; non-forest in 1990 but forest in 2000) but it is hardly visible because of the small amount detected. Note that the map is a mosaic of eight Landsat image pairs. The Greater Caucuses Mountain Range stretches along the Russian border.

**Figure 3:**
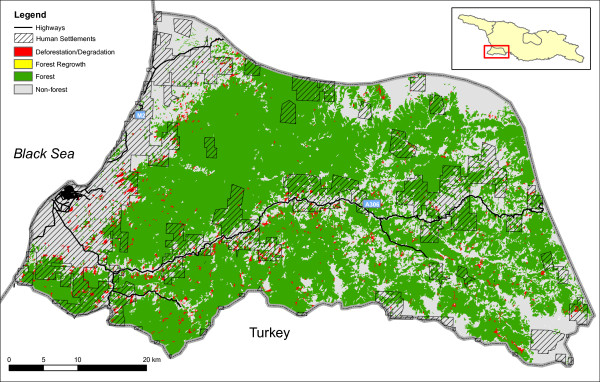
**Adjara changemap**. As Figure 2 but for autonomous republic of Adjara, 1987-2000. The map also shows major highways and areas of human settlement. The Adjaristsqali River flows along the A306 highway. Road data was obtained from OpenStreetMap (^©^OpenStreetMap contributors, CC-BY-SA) and data of human settlement was derived from NOAA's Nighttime Lights Time Series.

The remote sensing analysis showed that the overall forest loss in Georgia between 1990 and 2000 was 0.82%, i.e 0.82% of the forest present in 1990 had been cut by 2000. Much of the loss is concentrated in the western part of the country. There is little change east of longitude 44°E and along the Greater Caucasus mountain range in the North (Figure 2). The breakaway regions, Abkhazia and South Ossetia which have not been under full government control since the beginning of the 1990 s, show little evidence of forest loss - 0.19% and 0.26%, respectively. Adjara (an autonomous republic of Georgia in the southwestern part of the country) exhibits a higher change rate than the rest of the country (2.36%). Figure 3, a change map for Adjara 1987-2000, shows that the logging is concentrated in areas near human settlements, mainly the forest bordering the coast, and along the Adjaristsqali River (which flows along the A306 highway). Data on human settlements were obtained from remote sensing of nighttime lights [19].

Only a very small amount of forest regrowth was found, 0.09% of the forest present in 1990, or about 200 ha a year. This rate was included as input to the model but excluded from the accuracy assessment because of the small number of regrowth polygons. Including or excluding the regrowth has a minor effect on the current carbon sink (less than 0.01 Tg difference).

The results of the accuracy assessment of forest change are shown in Table 1. The areas of the land use classes were adjusted on the basis of the class accuracies as described in the section "Confidence Intervals and Area Adjustment". The percentage change dropped from 0.82% to 0.80%. The standard error for the change area was 9,520 ha with the 95% confidence interval ranging from 3,331 to 41,408 ha (the total area of Georgia is 6,970,000 ha). The adjusted area of the *stable forest *class was 41%, which is close to the official estimate (40%) of forest cover from the Forestry Department [20].

**Table 1:** Error matrix for the classified Landsat scenes*.

*h*	*1*	*2*	*3*	***n***_***h***_	***A***_***h***_	***W***_***h***_	*ϕ*
*1*	51	23	13	87	22,044	0.0032	0.0033
*2*	0	416	15	431	2,694,787	0.397	0.41
*3*	1	20	410	431	4,071,576	0.600	0.58
Total	52	459	438	949	6,788,387	1	1

*h*	*A*_*h, a*_	*S*(*A*_*h, a*_)	*Lower CI*	*Upper CI*	*User's*	*Prod's*	*Prop*.

*1*	22,370	9,519	3,331	41,408	59	98	0.19
*2*	2,795,765	47,690	2,700,384	2,891,146	97	91	38
*3*	3,970,273	48,528	3,873,217	4,067,328	95	94	57

	*Overall accuracy*	*Area-weighted accuracy*					

	92	96					

*Map categories are rows (1 = *forest-to-non-forest*; 2 = *stable forest*; 3 = *stable non-forest*) while the true categories are in columns. *h *is the map category; *n_h _*is the number of samples in *h*; *A_h _*is the area of map category *h*, with *A_h, a _*being the adjusted value of *A_h_*; *W_h _*is the proportional area of *h*; *ϕ *is proportion of samples in map category *h*; *S*(*A_h, a_*) is the standard error of the adjusted area; *Lower/Upper CI *are the upper and lower confidence intervals; *User's *and *Prod's *are user's and producer's accuracy in percent; *Prop*. is the user's accuracy weighted by map category area proportion. *Overall accuracy *and *Area-weighted accuracy *are expressed in percent. Area and confidence intervals are expressed in hectares.

Despite the high overall accuracy and area-weighted accuracy, the *forest-to-non-forest *class shows relatively low accuracy. However, the area estimate for this class changes very little when the information in the error matrix is taken into account. This result occurs because the many errors of commission in the *forest-to-non-forest *category are offset in area by the single error of omission from the *forest-to-non-forest *class. That single error is an error of commission in the *stable non-forest *class, and, given the large area weight, it offsets the more numerous errors of commission in the *forest-to-non-forest *class.

### Carbon Modeling

**Figure 4:**
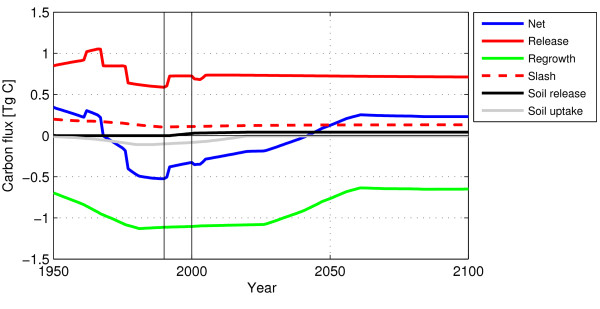
**Georgia carbon fluxes**. Carbon flux as an effect of land use change. "Slash" is the release of carbon from decaying slash left on the site after harvest or clearing; "Soil release/uptake" is the carbon released/accumulated from the soil following clearing of forest/afforestation; "Release" is the carbon released from fuelwood and decaying wood products; "Regrowth" is the carbon uptake from regrowing trees. The two verticale lines show the time period covered by the remote sensing analysis.

The carbon model was run using our best estimates for all rates - we used the remotely sensed estimate of deforestation, and the harvest and afforestation rates provided by the Forestry Department. For the future, we assumed that the 1990-2000 rates remain constant. As shown in Figure 4, the model results show that Georgian forests are currently a carbon sink of about 0.3 Tg C/y (0.35 Tg in 2004; 0.26 in 2010). Georgia will remain a sink with the magnitude slowly declining to zero by about 2040. The sink in 2004 is equivalent to 31% of the anthropogenic emissions [21].

To test the sensitivity of our results to various input parameters, the model was run with the illegal logging rate provided by the Forestry Department, again assuming that it will persist in the future. This change results in a larger sink which will not turn into a source until around 2060. Using these low rates of illegal logging, the sink is 47% of the anthropogenic emissions [21].

**Figure 5:**
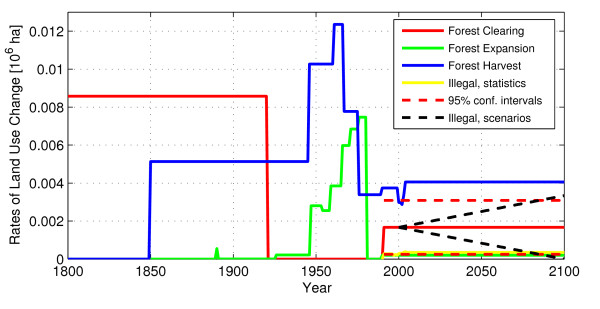
**Input land use rates**. The rates used as input to the book-keeping model. "Illegal, scenarios" refers to two scenarios of illegal logging: a doubling/drop to zero in 2100 of the rate observed from satellite. "Illegal, statistics" refer to the statistics provided by the Forestry Department. The 95% confidence intervals are of the forest clearing estimated by remote sensing (solid red line, 1990-2000).

**Figure 6:**
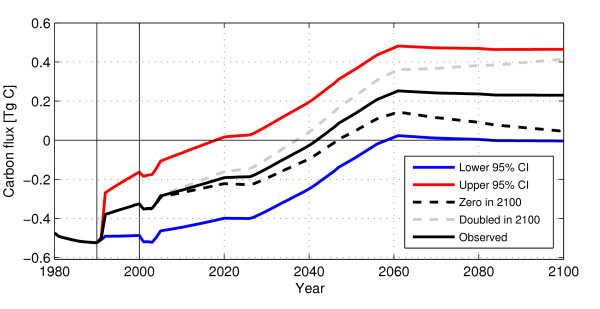
**Georgia carbon fluxes, scenarios**. Net carbon flux for different scenarios of illegal logging. "Lower 95% CI" refers to the lower 95% confidence interval of the remote sensing estimate; "Upper 95% CI" refers to the lower 95% confidence interval of the remote sensing estimate; "Zero in 2100" refers to a linear decrease from current rate to no illegal logging in 2100; "Doubled in 2100" refers to a linear increase from current rate to current rate times two in 2100; "Observed" means current illegal rate as observed from satellite persist. The two verticale lines show the time period covered by the remote sensing analysis.

One of the strengths of the model is the ability to explore the effect of different land use scenarios. Instead of assuming the the logging rates remain constant, the model was run assuming that the observed rate linearly (i) decreases to zero in 2100, and (ii) increases to double the observed rate in 2100 (other rates held constant). Scenario (i) will result in a carbon source around 2050, but the forests will again turn into a sink by the turn of the century; (ii) will accelerate the net carbon release and increase the source strength throughout the century. Input to the model is shown in Figure 5 and the resulting net fluxes in Figure 6.

## Discussion

### Change Analysis

The area that has changed from forest to non-forest is relatively small, about 0.3% of the total area, making the area adjustment sensitive to errors in the classification. Although the adjusted area is close to the original area (about 300 ha difference), the change area would have increased by almost 10,000 ha if one more of the *stable non-forest *samples had been forest change. The change area would almost double if two more *stable non-forest *samples were errors of omission from the change class. Classifying the non-change classes correctly - *stable forest *and *stable non-forest *- is even more important than getting the change class right. The sensitivity of the change class is reflected in the confidence intervals: with a 95% probability, the change area is within the interval of 3,331 to 41,408 ha (22,370 ± 19,038 ha) even though the overall accuracy of the classification is 92%. Such a variation in the estimated logging rate will propagate to the final carbon flux estimate and generate very different estimates of sink strength - the lower interval results in a net carbon source less than 0.1 Tg per year during the later half of the 21st century, while the upper interval results in a carbon source of around 0.5 Tg per year for the same time period. In comparison, using the adjusted remotely sensed logging estimate gives a source of 0.3 Tg per year (Figure 6). This highlights the importance of the accuracy assessment and the required adjustments.

We refer to the observed logging rate as "illegal" for reason stated in the Methods section below. However, it is likely that some forest has been cut or lost due to events such as fires, land slides, or infrastructural expansion during the 1990's. If the observed forest loss is assumed permanent (i.e land use change), the distinction between these changes and illegal logging do not matter from the perspective of carbon accounting.

The error matrix in Table 1 reveals that a relatively high number of polygons classified as change turned out to be stable forest. The main reason is the nature of the logging - there are very few clear cut areas. Instead, the typical logged area is only partially cut with some intact trees present in the polygon. Hence, changes in the images before and after trees have been cut are not as obvious as when an area has been clear cut. We defined an area to have been logged if half of the trees had been cut, however, determining the percentage cut is challenging. When available, high resolution imagery in GoogleEarth™was used for evaluating our accuracy assessment sites to determine if they had lost more than half their forest cover. In other cases we relied on the spectral signatures of the Landsat data.

This pattern of smaller areas of partial harvest suggests that illegal logging on an industrial scale did not take place, at least not between 1990 and 2000. Instead, the logged wood is most likely for household use. Further support for this finding is given by the fact that the logged areas usually are in close proximity to villages and accessible by roads, and that 54% of the Georgian households rely on wood for cooking [18]. Georgia seems to differ from other post-communist countries such as Russia and Ukraine, where wood destined for foreign export was illegally logged after 1990 [7,14].

As evident in the error matrix, detecting small scale logging from Landsat data is problematic. With a minimum mapping unit of 1 ha, we will miss some of the small change areas. Fuel wood extraction may selectively target individual trees and only degrade forests. Furthermore, the model uses area estimates of conversion of forest to non-forest. As the observed change area typically is a result of a degradation of the forest cover rather than complete deforestation, we are likely to over estimate the amount timber volume logged per unit area. We therefore run a risk of underestimating the amount of forest loss as the extraction of wood may take place at a scale not detectable by Landsat data; and other hand, we run a risk of overestimating the forest loss as the observed areas are often only partially cleared. The amount of over-/underestimation is unknown but a simple sensitivity analysis can be performed to assess how changes in the observed forest loss affect the carbon flux. Varying the observed logging rate ± 15% shifted the current (2010) carbon sink +12% and -17%, respectively; varying the rate ± 25% shifted the sink +22% and -27%. Changes in the observed logging rate thus shifts the carbon sink strength proportionally. How large the error marginals in the estimation of logged forest are, and if they compensate each other, needs further investigation.

The breakaway regions, Abkhazia and South Ossetia, which have been partially outside governmental control since the beginning of the 1990 s, and thereby outside the control of the Georgian Forestry Department, do not exhibit rates of logging higher than the rest of Georgia. These areas seem to follow the general pattern of low change rates in areas of mountainous, inaccessible terrain and low population densities. A higher logging rate was found in Adjara (Figure 3) which most likely can be attributed to a higher population density (twice as high as the Georgian average [22]) and a more accessible landscape compared to the northern part of the country.

### Land Use Related Carbon Sink

Figure 4 shows the net flux of carbon as a consequence of changes in land use from 1950 to 2100. The current sink is mainly a result of the small amount of forest being cleared during the 20th century (prior to 1990) and of trees regrowing following a relatively high rate of forest harvest prior to the 1960 s. The latter is reflected in the current age distribution with between 35% and 40% of Georgian forests between 41-60 years old (Table 2; [20]). During the 1960 s and 70 s, the harvest rate decreased rapidly; afforestation activities came to a halt around 1980, and logging increased following independence in 1991. These factors will eventually lead to the current sink becoming a source. It is probable that the magnitude and length of the source will be determined by the illegal logging rate as the harvest and afforestation activities are less likely to change in the future.

**Table 2:** Age class distribution from 2006.

*Age [years]*	*Growing years*	*Percent of forest*
1-20	1986-2005	2%
21-40	1966-1985	6%
41-60	1946-1965	37%
61-100	1926-1945	17%
101-	1925-	38%

Illegal logging significantly influences the terrestrial carbon budget of Georgia (Figure 6). There are dramatic differences possible as a result of different rates of logging. Figure 6 also shows the net carbon flux using the rates on illegal logging provided by the Forestry Department. The carbon sink for 2004 is almost 20 percent higher using the official estimate than if using the remotely sensed estimate, highlighting the discrepancies between the official numbers and our analysis. We have not found any studies presenting estimates on the carbon budget of Georgia.

What is the most probable scenario for the future? Will the logging rate increase, decrease or remain constant? After independence, Georgia experienced great economic turmoil and in 1994, 65% of the GDP came from the agricultural sector, a share that around 2000 had sunk to pre-independence levels [16]. This together with the fact that more than 50% of the Georgian households rely on wood for cooking, compared to 1.2% of the households in the Tbilisi region [18], may suggest a return to rural environments and in turn, increased demand for fuel wood after 1990. As the economy grows (Georgia's GDP grew more than 12% in 2007 [16]), less reliance on fuel wood could be expected.

However, the proportional increase of the agricultural contribution to the GDP after 1990 is the result of contraction of the industrial and service sectors. This claim is supported by the fact that the overall GDP per capita decreased by more than half between 1990 and 2000. And although the share of the population living in rural areas increased during this time period, both total and rural population decreased dramatically with the decrease in rural areas being slightly less [16]. As such, there is little evidence to support the claim of a population shift from urban to rural areas after 1990.

Georgia is a close ally to the European Union and a presumptive future member [23]. Projects such as the Protected Areas Development Project, financed by The World Bank, are aimed at conserving the Georgian biodiversity [24]. The combined effect of these projects and economic growth may result in lower rates of deforestation. On the other hand, as the economy develops, the anthropogenic emissions are likely to increase may weaken the net effect of the terrestrial carbon sink even though the deforestation would decrease.

The total amount of land area omitted because of clouds was 190,000 ha (2.7% of the total area). A large part of this area is located in the central, southern and non-forested parts of the country (Figure 2). Other areas are to a large extent forested. As the model is run using area estimates of land use change rates, any change area covered by clouds will be excluded from the analysis. If assuming that half of the omitted area is forested and that the proportion of land use change is the same as for the rest of country (0.8%), we would miss about 50 ha/year. This would have a minor effect on the net carbon flux, equivalent of decrease of the sink strength of less than 3% in 2010.

## Conclusions

The collapse of the Soviet Union brought dramatic political and economic changes to Georgia and many other countries. The removal of centralized control of the forest industry combined with increased reliance on wood for cooking and heating has resulted in industrial scale illegal logging in other part of the former Soviet Union [7,14,15]. For Georgia however, this does not appear to be the case. We found that the rate of deforestation is low with 0.8% of the forest cut between 1990 and 2000. We assume that the observed change is mainly a result of illegal logging, but we have not found any evidence of large-scale clear-cutting. Instead local harvesting of timber for household use is the underlying driver of the observed logging. The observed rates though are higher then the officially reported values, and highlighting the importance and need of remote sensing for monitoring land use change, deforestation in particular.

The current rate of deforestation with the history of Georgian land use make Georgian forests a carbon sink of 0.3 Tg of carbon per year which corresponds to 31% of the anthropogenic carbon emissions in 2004. Assuming that the current forestry activities do not change, the illegal logging rate is the main determinant of the magnitude of the future carbon sink. With the current deforestation rate, Georgian forests will become a source around 2040 but if the rate is decreases to zero in 2100, Georgian forests will become a carbon sink again in the future.

If the recent economic growth continues, it is likely that the illegal logging will decrease as the reliance on wood for fuel declines. Future monitoring of Georgian forests by remote sensing is recommended - a task that has been made easier with the opening of the Landsat archive [25] and the planned Landsat Data Continuity Mission [26].

## Methods

### Study area

Georgia is located by the Black Sea in Southwestern Asia. It borders the Russian Federation to the North, Turkey and Armenia to the South, and Azerbaijan to the East (Figure 1). With its proximity to the Black Sea, and with the Caucasus Mountains stretching across the country, the Georgian landscape is highly variable and includes temperate rain forests, alpine and semi-arid regions, deciduous and coniferous forests. Climatic conditions vary from a humid subtropical climate along the coast to boreal conditions high in the mountains and semi-arid regions in the East. The mean monthly temperatures range from 3°C in January to 26°C in July, and the annual precipitation is 462 mm [27]. It has an area of 70,000 km^2 ^with 40% of the area covered by forests [20].

### Remote Sensing

Landsat images from circa 1990 and 2000 were acquired over Georgia to estimate changes in forest cover. We processed the eight Landsat scenes that cover the entire country (Figure 1). Sensors and acquisition dates for the imagery are given in Table 3. All images are freely available for download [28] and were orthorectified prior to delivery. The spatial resolution of the data is 28.5 m and each scene covers an area of 185 × 185 km.

**Table 3:** Acquisition dates and satellite sensor systems used in the remote sensing analysis.

*WRS*		*First acquisition*		*Second acquisition*	
***Path***	***Row***	***Satellite System***	***Date***	***Satellite System***	***Date***

169	31	Landsat 4 TM	Aug 8 1989	Landsat 7 ETM+	Aug 20 1999
170	30	Landsat 4 TM	Aug 31 1989	Landsat 7 ETM+	Jun 13 2001
170	31	Landsat 4 TM	Aug 31 1989	Landsat 7 ETM+	Jun 13 2001
171	31	Landsat 4 TM	Sep 23 1989	Landsat 7 ETM+	Sep 5 2000
171	30	Landsat 5 TM	Sep 26 1987	Landsat 7 ETM+	Jun 17 2000
172	31	Landsat 5 TM	Aug 16 1987	Landsat 7 ETM+	Jul 10 2000
172	30	Landsat 5 TM	Sep 28 1991	Landsat 7 ETM+	Sep 12 2000
173	30	Landsat 5 TM	Sep 24 1987	Landsat 7 ETM+	Sep 06 2001

Change in forest cover was detected using a supervised neural network classifier [29,30]. Changes in brightness, greenness and wetness [31] between the two time periods were used as input to the neural network together with the brightness, greenness and wetness transformation of the second date of image [32]. All imagery was atmospherically corrected using dark object subtraction. The results include the classes of *forest loss *and *forest regrowth*, *stable non-forest*, *stable forest*; and *snow*, *clouds *and *shadows *(Table 4). Training areas were used to train the classifier, and the methodology is described in [33]. The neural network classifier assigns a land cover class to each individual pixel which often results in scattered single misclassified pixels. A segmentation algorithm [34] that groups neighboring pixels into polygons, with 1 ha minimum mapping unit, was applied to the output to remove scattered pixels and define polygons that more realistically correspond to landscape features. The resulting polygon-based maps were manually inspected and misclassified polygons were relabeled.

**Table 4:** Land cover classes; date 1 and 2 refers to the first and second image acquisition for same scene.

*No*.	*Name*	*Description*
1	Change	Forest present date 1 but not date 2
2	Regrowth	Forest present date 2 but not date 1
3	Con. forest	Coniferous Forest present date 1 and 2
4	Dec. forest	Deciduous forest present date 1 and 2
5	Non-forest	No forest present date 1 and 2
6	Clouds 1	Clouds present date 1 but not date 2
7	Shadows 1	Shadows present date 1 but not date 2
8	Clouds 2	Clouds present date 2 but not date 1
9	Shadows 2	Shadows present date 2 but not date 1
10	Snow	Snow present either date

Areas of clouds and shadows were omitted from the analysis. The total area omitted was approximately 190,000 ha or 2.7% of the total area. Areas covered by snow and ice was classified as *stable non-forest*.

### Confidence Intervals and Area Adjustment

The accuracy of the classification was assessed by 949 random samples, each consisting of a polygon created by the segmentation algorithm. Three map categories were considered: (1) change from forest to non-forest, (2) *stable forest *and (3) *stable non-forest*; forest regrowth was excluded because of the low number of regrowth polygons. The samples were stratified by map category - 431 samples were allocated to the category 2 and 3, and 87 to category 1.

The results of the accuracy assessment are shown in Table 1 in form of a confusion matrix. From the confusion matrix it is possible to estimate the overall map accuracy (92%) as well as an area-weighted accuracy (96%). The area-weighted accuracy is necessary because of the stratified sample design that allocated a disproportionately large number of samples to the forest to non-forest class. It is also possible to calculate the accuracy of the individual classes from both the user's and producer's perspective (Table 1).

While the accuracy of the map is important, is is the area estimates of the class, and the forest to non-forest class in particular, that is central to this study.

The simplest way to estimate the area of the forest to non-forest class is to count the pixels in that class in the map. But the accuracy assessment provides us additional information that can be used to adjust the area estimates on the basis of what is learned in the accuracy assessment.

With the confusion matrix and the map area of the different map categories (Table 1), it is possible to calculate the 95% confidence intervals and an adjustment factor for the estimated area of any map category. The error matrix reveals if each map category is over or under represented in the map. With this information, it is possible to obtain adjusted area estimates of the different categories. Here we used the equations in [35] but this analysis can also be performed using the equations in [36] which give a very similar result.

The adjusted area (*A*_*h, a*_) is obtained by multiplying the observed area (*A_h_*) by the proportion of samples in map category *h *(ϕ). In this case, the map category of interest is the forest to non-forest category (*h *= 1).

(1) $$A_{h=1,a}=A_{h=1}\times \phi ;$$

(2) $$\phi =\sum_{h}W_{h}\times p_{h},$$

where *W_h _*is the proportional area of *h*, and *p_h _*is the proportion of correct samples in the forest to non-forest category to the total number of samples in *h *(*n_h_*).

The standard error of adjusted change area, *S*(A_*h*_), is given by

(3) $$S(A_{h=1})=A_{tot}\times S(\phi ),$$

where *A_tot _*is the area of the all map categories. The confidence intervals (*CI*) are calculated as

(4) CI=±2S(ϕ),

and the estimated variance and standard error of *ϕ *as

(5) $$V(\phi )=\sum_{h}\frac{W_{h}^{2}\times p_{h}\times (1-p_{h})}{\left(n_{h}-1\right)};S(\phi )=\sqrt{V(\phi )}.$$

### Carbon Modeling

As humans alter the landscape, carbon can be either released to, or withdrawn from, the atmosphere. The main changes in land use associated with significant fluxes of carbon are harvesting of forests, reforestation or afforestation, and clearing of forest for agricultural lands and pasture. Harvested wood will release carbon back to the atmosphere, either immediately through burning or over time as wood products oxidize through decay. In addition, carbon is released from the soil as organic matter decays, and from decaying slash left on the site following forest harvest (see e.g. [37,38]). Similar losses of carbon from the biosphere occur when forests are converted into agricultural lands. If reforestation follows harvest, carbon will again be accumulated in both vegetation and soil.

To quantify the response of an ecosystem to changes in land use, it is necessary to track changes in the terrestrial carbon pools over time based on known rates of land use change. Thereby the net exchange of carbon between the atmosphere and the biosphere can be estimated (see e.g. [37,38]). The carbon book-keeping model employed in this study has been used to estimate the effect of land use change on terrestrial carbon fluxes since the beginning of the 1980 s (see e.g. [11,37-40]). The term "book-keeping" stems from the fact that the model tracks carbon stocks from year to year rather than trying to model the individual biological processes that constitute the carbon cycle, i.e. photosynthesis and respiration [37]. As a consequence, it is not possible to verify the model results with direct atmospheric measurements such as flux tower measurements. Furthermore, there is no attempt to include inter-annual variability in forest growth. Instead, the model makes use of forest harvest and clearing estimates and average forest growth rates; and takes into account the time lags associated with decomposition of wood products. It also takes the depletion/accumulation of soil carbon into account. For the above-mentioned reasons, the model is well suited for estimating the effects of land use change on terrestrial carbon budgets on a national scale over time periods ranging from years to decades. The use of remote sensing to provide rates of land use change obviates the need for recent times to rely on the uncertain national estimates reported to FAO (see e.g. [4,11]).

In Georgia, three kinds of land use change were considered: (i) harvest of forest (reforestation assumed); (ii) clearing of forest for agriculture or pasture; and (iii) afforestation or natural reforestation on abandoned land. These rates of land use change, expressed as a time series in hectares per year, are used as input to the model. Each change can be regarded as an event, or disturbance, in the ecosystem generating a response expressed in terms of carbon being released or accumulated. The sum of these responses gives the final carbon flux. The model estimates the uptake and release (and net flux) at an annual time step.

Below is a description of the different kinds of events and the associated ecosystem responses.

#### Event: Forest Harvest

For this category of event, forest is harvested at time to, with reforestation occurring afterward. Part of the harvested wood will be used as firewood and will be released back into the atmosphere within a year after harvest. Another part will be used for short-lived wood products, like paper, assumed to decay at a rate of 10% per year. The final and third part will end up as long-lived wood products, such as furniture and building materials, which are assumed to decay at a rate of 1% per year. These three pools are referred to as the 1, 10 and 100 year pools. Accordingly, the harvested wood is distributed among these three different carbon pools with three different decay rates; and the amount and timing of carbon released as a consequence of the harvest event is determined by the amount of harvested wood and its distribution among the pools [38]. In addition to the above, carbon is released from slash left at the scene after harvest, resulting in a fourth source of carbon release.

Since the harvest is followed by reforestation, carbon will be sequestered in the regrowing trees. The rate of sequestration is based on average growth rates for the region as a function of the age of the forest. The regrowing forest will reach a point where the growth is sufficient to allow for a second harvest and another event may take place.

Equation 6 sums the different carbon sinks and sources of a forest harvest event (release is positive, or as a "source").

(6) $$\begin{array}{c} Flux_{harvest}\;=\;Pool_{1y}\;+Pool_{10y}\;+Pool_{100y}\;+\\ Slash-Regrowth. \end{array}$$

**Figure 7:**
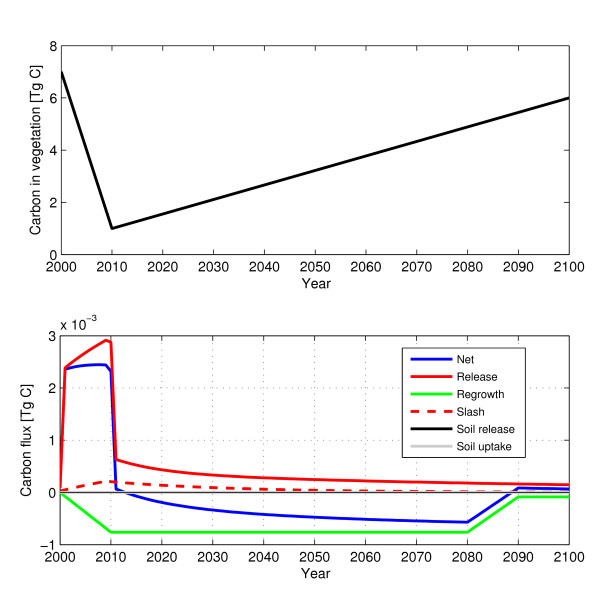
**Harvest event**. The carbon release and uptake associated with harvest over a ten year period. (Forest replanted after harvest. 50 km^2 ^harvested annually between 2001 and 2010.). The top figure shows the carbon stored in vegetation through time. The bottom graph shows the individual pools of carbon through time.

Figure 7 shows the carbon release/uptake of an ecosystem over time following a harvest event.

#### Event: Forest Converted to Cropland/Pasture

When a forest is cleared for agriculture or pasture, the ecosystem will lose carbon stored in both the soil and vegetation. The latter is treated the same as the forest harvest case - the wood material is assumed to end up in the three carbon pools and slash, and will decay at different rates accordingly. But since the cleared forest does not regrow trees, there will be no carbon uptake from regrowth of new trees.

Following conversion from forest to agriculture or pasture the system will experience a loss of soil carbon. The loss is exponential with a sharp increase in the release following the clearing [38].

Equation 7 sums the sources of an ecosystem in the event of forest clearing (release is positive).

(7) $$\begin{array}{l} Flux_{clearing}\;=\;Pool_{1y}+Pool_{10y}+Pool_{100y}+\\ \;\;Soil_{release}+Slash. \end{array}$$

#### Event: Afforestation or Abandonment of Cropland/Pasture

When a cropland or pasture is abandoned, trees start growing, resulting in carbon being sequestered. When trees start growing, soil organic matter starts to accumulate, adding to total carbon uptake. Hence, there is no release of carbon associated with the event of abandonment of agricultural fields [38]. The same holds true for afforestation.

Equation 8 sums the sinks of an ecosystem in the event of afforestation/abandonment of cropland.

(8) $$Flux_{abandon}=-Regrowth-Soil_{uptake}.$$

### Input for the Carbon Model

Historical rates of land use change used in the model were provided by the Georgian Forestry Department. Data on forest harvest were given as an annual time series dating back to 1949 of the amount of timber volume officially harvested. This harvest is part of the forest management activities conducted by the Georgian Forest Department. There is no clear-cutting of forest as only individual trees are cut. The forest is allowed to naturally regenerate. Since only individual trees are being cut, it is hard, if not impossible, to detect this activity from space.

Afforestation activities in Georgia commenced during the end of the 19th century. Annual afforestation rates for 1890-1980 provided by the Georgian Forestry Department were used as input for the third type of land use change.

Figure 5 shows the rates of forest change over time. The large increase in forest clearing starting in 1800 was included to reflect the decrease in forest cover from about 55% during the 19th century to 41% in 1921 when Georgia became part of the Soviet Union. We have little information on how and exactly when this change in land use took place. Furthermore, we have data on forest harvest from 1949 and onwards, however, harvest activities commenced earlier and pre-1949 rates were reconstructed to reflect the current (2006) age class distribution. We know that 37% of the Georgian forests are 41-60 years old and 38% are above 100 years, whereas 17% are 61-100 years [20]. The pre-1945 harvest rate was therefore set to half of the 1949 rate. It could be argued that the rate prior to 1906 should be increased to reflect the high percentage of trees older than 100 years, but the 19th century forest harvest rate is likely to have been less than that of 1949. It is reasonable to believe that the Georgian forests sustained less people during the 19th century than during the mid of the 20th century. Technological progress is also bound to have increased the harvest since the 19th century. We therefore did not increase the rate prior to 1945 but kept the same rate back to 1850. The 19th century harvest rate has little effect on the current carbon budget though.

The rate of illegally cut forest was used as observed via remote sensing to estimate the rate of clearing of forests for pasture. Furthermore, it is assumed that the illegal activities began as Georgia gained independence from the Soviet Union in 1991 as reported by the Forestry Department. This assumption follows extensive discussions with forest managers and is based on the fact that wood became the main source of household fuel as delivery of natural gas drastically declined after independence. Although only single trees were being cut, illegal harvest activities usually resulted in coherent areas being affected. The cutting tends to take place near villages where grazing areas are expanded at the expense of forest. Two sources for the rate of illegal logging for the model were used: (i) Official statistics from The Georgian Forestry Department. Annual values were provided from 1995 to 2005 (the cutting rate of 1995 was assumed to represent the period 1991-1994). (ii) Forest change rates observed from satellite. The Forestry Department does not clear cut forests whereas the illegal logging results in complete or partial clearing. Thus, it is assumed that change areas detected from satellite are a result of illegal activities. The change rate derived from the remote sensing analysis multiplied by the average biomass was taken as an estimate of the amount of illegally logged forest between 1990 and 2000 (which was assumed to persist after 2000).

Coefficients for the response functions describing the uptake/release of soil carbon following afforestation/clearing were taken from [20,41]. If we were unable to a find a reliable value for any of the parameters we used data from a carbon budget study for Romania (unpublished results in preparation). The list of all coecients is presented in Table 5.

**Table 5:** Values of model coefficients. "h" stands for harvest and "c" for clearing.

*Coefficient*	*Value*	*Source*
Slash ratio following clearing	0.33	[41]
Slash ratio following harvest	0.09	Value for Romania
Slash decay rate	0.04	[41]
Fraction of C assigned to 1 year pool (c)	0.500	Value for Romania
Fraction of C assigned to 10 year pool (c)	0.100	Value for Romania
Fraction of C assigned to 100 year pool (c)	0.070	Value for Romania
Fraction of C assigned to 1 year pool (h)	0.307	[20]
Fraction of C assigned to 10 year pool (h)	0.072	[20]
Fraction of C assigned to 100 year pool (h)	0.531	[20]
C content of mature forest [t/ha]	144	Value for Romania
Minimum C content after disturbance [t/ha]	5	Value for Romania
Initial C content of disturbed system [t/ha]	127	Value for Romania
Initial recovery time after disturbance [y]	80	Value for Romania
Full recovery time after disturbance [y]	100	Value for Romania
Soil C content in undisturbed systems [t/ha]	134	[41]
Soil C content after disturbance [t/ha]	114	[41]
Minimum soil C content [t/ha]	107	[41]
Recovery time for soil C after abandon. [y]	40	[41]

## Competing interests

The authors declare that they have no competing interests.

## Authors' contributions

PO drafted the manuscript and carried out the remote sensing and carbon modeling analysis; CEW, AB, MO and PO conceived the study. AB assisted in the remote sensing analysis. PO and PT obtained the data for the model and carried out the main field study. PO, CEW and MO did additional field studies. FZ, XY, RAH, CEW and PO made a new implementation of the carbon book-keeping model. All authors read and approved the manuscript

## References

[B1] IPCC Fourth Assessment Report. Climate Change 2007: Synthesis Report 2007

[B2] IPCC Fourth Assessment Report. Climate Change 2007: The Physical Science Basis 2007

[B3] FRA Global Forest Resources Assessment 2005 FAO, Rome 2006 [FAO Forestry Paper 147]

[B4] Hansen MC Stehman SV Potapov PV Loveland TR Townshend JRG DeFries RS Humid tropical forest clearing from 2000 to 2005 quantified by using multitemporal and multiresolution remotely sensed data Proc Natl Acad Sci 2008 105 9439 9444 10.1073/pnas.0804042105 18591652 PMC2453739

[B5] UN-REDD UN Collaborative Programme on Reducing Emissions from Deforestation and Forest Degradation in Developing Countries (UN-REDD). FAO, UNDP, UNEP Framework Document 2008

[B6] FRA Global Forest Resources Assessment 2000 FAO, Rome 2001 [FAO Forestry Paper 140]

[B7] Kuemmerle T Chaskovskyy O Knorn J Kruhlov I Radeloff V Hostert P Forest cover change and illegal logging in the Ukrainian Carpathians in the transition period from 1988 to 2007 Remote Sens Environ 2009 113 1194 1207 10.1016/j.rse.2009.02.006

[B8] Grainger A Difficulties in tracking the long-term global trend in tropical forest area. Proc Natl Acad Sci 2008 105 818 823 10.1073/pnas.0703015105 18184819 PMC2206620

[B9] Li R Buongiorno J Turner JA Zhu S Prestemon J Long-term effects of eliminating illegal logging on the world forest industries, trade, and inventory Forest Policy and Economics 2008 10 480 490 10.1016/j.forpol.2008.04.003

[B10] Rudel TK Coomes OT Moran E Achard F Angelsen A Xu JC Forest transitions: towards a global understanding of land use change Global Environmental Change-Human and Policy Dimensions 2005 15 23 31 10.1016/j.gloenvcha.2004.11.001

[B11] DeFries R Houghton RA Hansen M Field C Skole DL Townshend J Carbon emissions from tropical deforestation and regrowth based on satellite observations for the 1980 s and 90 s Proc Natl Acad Sci 2002 99 22 14256 14261 10.1073/pnas.182560099 12384569 PMC137871

[B12] The World Bank Forests Sourcebook 2008 Washington DC: 2008 The International Bank for Reconstruction and Development/The World Bank

[B13] Bouriaud L Causes of illegal logging in Central and Eastern Europe Small-scale Forest Economics, Management and Policy 2005 4 269 292

[B14] WWF Illegal Logging in the Southern Part of the Russian Far East [World Wildlife Fund Russia] 2002

[B15] Newell J Levedev A Plundering Russia's far eastern taiga: illegal logging, corruption and trade 2000 [Bureau for Regional Oriental Campaigns and Pacific Environment Resource Committee, Vladisvostok, Russia]

[B16] World Bank World Development Indicators 2010 http://data.worldbank.org

[B17] UNICEF Children And Women In Georgia: A Situation Analysis Tech. rep., United Nations Children's Fund 2003

[B18] State Department of Statistics Monitoring the situation of children and women: Multiple Indicator Cluster Survey 2005 Tech. rep., State Department of Statistics of Georgia, National Centre for Disease Control - Georgia, United Nations Children's Fund 2008

[B19] Elvidge CD Sutton PC Tuttle BT Ghosh T Baugh KE Global Mapping Of Human Settlement: Experiences, Datasets, and Prospects Boca Raton: CRC Press, Taylor and Francis Group 2009 chap. Global Urban on Mapping Based Nighttime Lights 129 145

[B20] Torchinava P Georgian Statistical Yearbook of Forestry 2006 [Forestry Depatment, Ministry of Environment Protection and Nature Resourcees of Georgia]

[B21] UN United Nations CO2 emissions estimates. Data from the UNSD Millennium Development Goals Indicators database 2007 http://unstats.un.org/unsd/environment/air_co2_emissions.htm

[B22] Ministry of Economic Development of Georgia Population Census 2002 Tech. rep., Ministry of Economic Development of Georgia, Department of Statistics 2009 http://www.statistics.ge/

[B23] European Commission European Neighbourhood Policy. European Union-Georgia Action Plan [Delegation Of The European Commission To Georgia] 2006

[B24] The World Bank Georgia - Protected Areas Development Project Tech. Rep. PID6578, The World Bank - Georgia 2001

[B25] Woodcock CE Allen R Anderson M Belward A Bindschadler R Cohen W Gao F Goward SN Helder D Helmer E Nemani R Oreopoulos L Schott J Thenkabail PS Vermote EF Vogelmann J Wulder MA Wynne R Free Access to Landsat Imagery Science 2009 320 1011 10.1126/science.320.5879.1011a 18497274

[B26] Loveland T Cochrane MA Henebry GM Landsat still contributing to environmental research Trends in Ecology and Evolution 2008 23 182 183 10.1016/j.tree.2008.01.002 18295369

[B27] Haggett P Encyclopedia of World Geography. Russia, Northern Eurasia 2002 Marshall Cavendish Inc

[B28] USGS USGS, Earth Resources Observation and Science (EROS). Global Visualization Viewer 2009 http://glovis.usgs.gov/

[B29] Carpenter GA Gjaja MN Gopal S Woodcock CE ART neural networks for remote sensing: vegetation classification from Landsat TM and Terrain Data IEEE Trans Geosci Remote Sens 1997 35 306 325

[B30] Carpenter GA Gopal S Macomber S Martens S Woodcock CE A neural network method for mixture estimation for vegetation mapping Remote Sens Environ 1999 70 135 152

[B31] Crist EP Cicone RC A Physically-Based Transformation of Thematic Mapper Data-The TM Tasseled Cap IEEE Trans Geosci Remote Sens 1984 22 256 263 10.1109/TGRS.1984.350619

[B32] Collins JB Woodcock CE An assessment of several linear change detection techniques for mapping forest mortality using Multitemporal Landsat TM data Remote Sens Environ 1996 56 66 77 10.1016/0034-4257(95)00233-2

[B33] Woodcock CE Macomber SA Pax-Lenney M Cohen WB Monitoring large areas for forest change using Landsat. Generalisation across space, time and Landsat sensors Remote Sens Environ 2001 78 194 203 10.1016/S0034-4257(01)00259-0

[B34] Woodcock CE Harward VJ Nested-hierarchical scene models and image segmentation Int J Remote Sens 1992 13 3167 3187 10.1080/01431169208904109

[B35] Cochran WG Sampling Techniques 1977 New York, NY: Wiley

[B36] Card DH Using map category marginal frequencies to improve estimates of thematic map accuracy Photogram Eng Remote Sens 1982 49 12 431 439

[B37] Moore B Boone RD Hobbie JE Houghton RA Melillo JM Peterson BJ Shaver GR Vorosmarty CJ Woodwell GM A simple model for analysis of the role of terrestrial ecosystems in the global carbon budget Modelling the Global Carbon Cycle, SCOPE Report No. 16 1981 New York: Wiley

[B38] Houghton RA Hobbie JE Melillo JM Moore B Peterson BJ Shaver GR Woodwell GM Changes in carbon content of terrestrial biota and soils between 1860 and 1980: a net release of CO2 to the atmosphere Ecol Modell 1983 53 235 262

[B39] Houghton RA The flux of carbon from terrestrial ecosystems to the atmosphere in 1980 due to changes in land use: Geographic distribution of the global flux Tellus 1987 39B 122 139 10.1111/j.1600-0889.1987.tb00277.x

[B40] Houghton RA Hackler JL Emissions of carbon from forestry and land-use change in tropical Asia Global Change Biol 1999 5 481 492 10.1046/j.1365-2486.1999.00244.x

[B41] Houghton RA Hackler JL Carbon Flux to the Atmosphere From Land-use Changes: 1850 to 1990 Tech. Rep. 131, NDP-050/R1, ORNL/CDIAC 2001

